# Fulfillment of the Brazilian Agenda of Priorities in Health Research

**DOI:** 10.1186/1478-4505-9-35

**Published:** 2011-08-31

**Authors:** Leonor Maria Pacheco Santos, Erly Catarina Moura, Rita de Cássia Barradas Barata, Suzanne Jacob Serruya, Marcia Luz da Motta, Flávia Tavares Silva Elias, Antonia Angulo-Tuesta, Ana Patricia de Paula, Gilvania de Melo, Reinaldo Guimarães, Carlos Augusto Grabois Gadelha

**Affiliations:** 1 Departamento de Saúde Coletiva, Universidade de Brasília. Campus Universitário Darcy Ribeiro, CEP 70910-900, Brasília, DF, Brazil; 2 Departamento de Ciência e Tecnologia, DECIT, Secretaria de Ciência, Tecnologia e Insumos, Estratégicos. Ministério da Saúde. Esplanada dos Ministérios bloco G, sala 849, CEP 70058-900, Brasília, DF, Brazil; 3 Departamento de Medicina Social, Escola de Medicina da Santa Casa. Rua Dr. Cesário Mota Junior 61, CEP 01221-020, São Paulo, SP, Brazil; 4 Latin American Centre for Perinatology and Women and Reproductive Health of the Pan American Health Organization (CLAP/WRH-PAHO). Avenida Itália, Hospital de Clinicas, Piso 16. Montevideo, CP 11100, Uruguay; 5 Faculdade de Ceilândia, Universidade de Brasília. QNN 14 Área Especial, Ceilândia Sul, CEP 72.220-140, Brasília, DF, Brazil; 6 Escola Superior de Ciências da Saúde da Fundação de Ensino e Pesquisa em Ciências da Saúde, Secretaria da Saúde, Governo do Distrito Federal. SMHN, Quadra 3, Conjunto A, Bloco 1, CEP 70710-907, Brasília, Brazil; 7 Instituto de Medicina Social, Universidade Estadual do Rio de Janeiro. Rua São Francisco Xavier, 524, Pavilhão João Lyra Filho, 7° andar/blocos D e E, Maracanã, CEP 20550-900, Rio de Janeiro, RJ, Brazil; 8 Secretaria de Ciência, Tecnologia e Insumos Estratégicos. Ministério da Saúde. Esplanada dos Ministérios bloco G, sala 802, CEP 70058-900, Brasília, DF, Brazil

## Abstract

This commentary describes how the Brazilian Ministry of Health's (MoH) research support policy fulfilled the National Agenda of Priorities in Health Research (NAPHR). In 2003, the MoH started a democratic process in order to establish a priority agenda in health research involving investigators, health managers and community leaders. The Agenda was launched in 2004 and is guiding budget allocations in an attempt to reduce the gap between scientific knowledge and health practice and activities, aiming to contribute to improving Brazilian quality of life. Many strategies were developed, for instance: Cooperation Agreements between the Ministry of Health and the Ministry of Science and Technology; the decentralization of research support at state levels with the participation of local Health Secretariats and Science and Technology Institutions; Health Technology Assessment; innovation in neglected diseases; research networks and multicenter studies in adult, women's and children's health; cardiovascular risk in adolescents; clinical research and stem cell therapy. The budget allocated by the Ministry of Health and partners was expressive: US$419 million to support almost 3,600 projects. The three sub-agenda with the higher proportion of resources were "industrial health complex", "clinical research" and "communicable diseases", which are considered strategic for innovation and national development. The Southeast region conducted 40.5% of all projects and detained 59.7% of the resources, attributable to the concentration of the most traditional health research institutes and universities in the states of São Paulo and Rio de Janeiro. The second most granted region was the Northeast, which reflects the result of a governmental policy to integrate and modernize this densely populated area and the poorest region in the country. Although Brazil began the design and implementation of the NAPHR in 2003, it has done so in accordance with the 'good practice principles' recently published: inclusive process, information gathering, careful planning and funding policy, transparency and internal evaluation (an external independent evaluation is underway). The effort in guiding the health research policy has achieved and legitimated an unprecedented developmental spurt to support strategic health research. We believe this experience is valuable and applicable to other countries, but different settings and local political circumstances will determine the best course of action to follow.

## Background

The establishment of a research priority agenda is important to ensure the best possible use of available resources, to identify the necessary resources against competing demands and to strengthen ties between policy, health practice, scientific knowledge and technological development [1]. The Global Forum for Health Research coined the term "10/90 gap" to capture the major imbalance in the allocation of health research funds, indicating that the magnitude of health problems is not proportional to the resources invested in addressing those [2].

The traditional mode of science production was based on universities and research institutes, with agenda set by investigators, where research was dichotomized as basic or applied, with a disciplinary approach. This is gradually replaced by a participatory mode; the new system is characterized by research networks, agenda defined in an application context, research aimed at problem-solving, transdisciplinary focus, assessment of academic merit and social relevance [3].

In this context, the formulation of a national agenda for health research must consider the population's level of development, demographic and epidemiologic profile, current level of knowledge and shortcomings, together with the diversity of regional situations, participation of public, philanthropic and private institutions, as well as intellectual property policies [4].

Research in Brazil gained magnitude, a fact acknowledged by the international press ("An emerging power in research; science in Brazil". *The Economist*, January 6th, 2011 <http://www.economist.com/node/17851421 >). However, up until recently, there was an incipient articulation between the Unified Public Health System (SUS) and the National Science and Technology System, which did not guarantee the application of results and findings to meet the SUS's social needs and purposes. To reverse this situation it was essential to bring the Ministry of Health to the center of research decisions, enabling integration between those who investigate, request, implement and use health knowledge.

The Department of Science and Technology (DECIT) at the Brazilian Ministry of Health (MoH) is responsible for promoting research to improve the health system. Its primary challenge is to guarantee scientific and technological development as an important and permanent tool for guiding the country to an evidence-based SUS that promotes universality, integrity and equity.

An essential step was establishing a National Agenda of Priorities in Health Research (NAPHR). The process started in 2003 when a Technical Advisory Committee composed of 20 distinguished scientists and policymakers was appointed. Shortly thereafter, a Seminar was organized with 510 professionals, among which health researchers (68%) and healthcare policymakers or providers (32%), who participated in the elaboration of the first NAPHR draft. The final choice regarding participants guaranteed a fair distribution as far as sex and place of origin, so that the country's five regions and most of the 27 states were represented [5]. The draft Agenda was submitted to public consultation on the website for 45 days when 1,900 individuals registered online; 360 comments and contributions were received and analyzed. The next step was very important to legitimate the process: the NAPHR was presented, discussed and approved during the National Conference [5] as described below.

In 2004 the Brazilian Ministry of Health fostered the organization of the 2nd National Conference on Science, Technology and Innovation (STI) in Health. As usual in National Health Conferences, a tradition in the SUS, the process was democratic with inputs from investigators, stakeholders and the public in general. It involved 15,000 participants in 307 local conferences, which indicated part of the 644 delegates for the National Conference. The delegates discussed and approved the National Policy on STI in Health and the NAPHR. The Policy is guided by six principles: (a) improving the Brazilian population's health conditions in the short, medium and long run; (b) overcoming all forms of inequity and discrimination (regional, social, ethnical, gender and others); (c) respect to people's lives and dignity; (d) ensuring implementation of high ethical standards in health research; (e) respect to methodological and philosophical plurality; (f) social inclusion, citizen control and respect to the environment and sustainability [6]. The Conference was a challenge for scientists, health providers and community leaders. They had never before interacted in such depth to openly discuss their points of view, not always an easy task, as pointed out by some authors [7].

Since then, research supported by DECIT has been guided by the NAPHR, which is organized into 24 sub-agenda according to life cycle, race, disease or damage and other criteria. The complete list of sub-agendas is available on the web site at the following address: <http://bvsms.saude.gov.br/bvs/publicacoes/agenda_ingles.pdf >[8]. Equally important to setting a National Priority Agenda is how it is implemented. This commentary intends to analyze how the Brazilian Ministry of Health research support policy fulfilled the 2004 NAPHR. Recently, there has been a greater recognition of the value of reviewing the research performed and/or the funding allocated based on previously established priorities [9].

### Institutional partnerships to implement the National Agenda of Priorities

An important step towards the rapid implementation of the NAPHR was the signing of Cooperation Agreements between the Ministry of Health (MoH) and the Ministry of Science and Technology (MST), from 2004 to 2012. This partnership involved the Financier of Studies and Projects (Financiadora de Estudos e Projetos, FINEP) and the National Council of Scientific Development (Conselho Nacional de Desenvolvimento Científico e Tecnológico, CNPq), both belonging to the MST. The agreements allow for a budgetary expansion to fund research and innovation projects, co-financed with the MST Health Sector Fund. State Foundations for Research Support (FAP) are also important MoH partners in the effort to implement the NAPHR, as we will explain in more detail in the following sections.

### Decentralization and democratization of health research

The infrastructure regarding research facilities, trained investigators, postgraduate courses and, consequently, financial resources, has always been concentrated in the Southeast, the richest and most developed region. In the nineties, this region used to attract the best scientists from all over the country in their search for better working conditions. This region received 48% of the 1,359 scientists who migrated internally from 1993 to 1999, when there was also an appreciable brain drain out of the country (n = 959) [10].

The MoH decided to foster health research in a decentralized way and initiated the project known as Research for SUS Program (PPSUS). In 2002/3 there were 147 research projects funded by this decentralized mechanism, resulting from public calls for proposals ran in 10 states. Considering the need to be more inclusive, the program was extended to all 26 Brazilian states, besides the Federal District, and in the next year, 2004, financed almost 400 projects. Since then, there are regular calls for proposals about every two years. The MoH, through DECIT, is the national coordinator of PPSUS and FAPs act as co-financiers and executing agents. The local FAPs and the state health departments are involved in all stages, from the selection of priorities to the judgment of proposals and the evaluation of final results.

The main objective of this initiative is to reduce regional inequities in scientific knowledge production. The accomplishment of this policy may be ascertained by the increased participation of state investments in health science and technology at each edition of the program.

The largest number of PPSUS projects was approved in the Northeast region (Table 1). However, the greatest sum of investment was allocated to the Southeast. This fact is related to some large vaccine development projects financed in the later, with large investments from the local FAPs.

**Table 1:** Proportion of projects approved and amount of resources allocated by the Ministry of Health and partners in the decentralized PPSUS program and academic degrees related to the projects, according to the geographic region, Brazil, 2004-2009.

Region	Number of PPSUS projects	Amount invested in PPSUS (U$ millions)	Proportion of Master + Doctor degrees (%)	Proportion of the Brazilian population (%)
Midwest	152	3.9	8.1	7.3
Northeast	581	14.3	32.1	28.0
North	236	9.8	7.6	8.0
Southeast	459	36.8	28.9	42.3
South	347	10.3	23.3	14.4
**Total**	**1,775**	**75.1**	**100.0**	**100.0**

Source: data summaries were calculated by the authors based on information taken from: http://www.saude.gov.br/sisct, and http://www.saude.gov.br/pesquisasaude, captured on Jan 21, 2011.

One indicator of PPSUS's success is scientific publications and qualification of human resources: 668 Masters Dissertations and 332 Doctoral Theses received support from the program and 25 patents were registered. The Northeast region reported the largest number of academic degrees, 322 Master and Doctoral degrees (corresponding to 32%), ahead of the most developed and populated Southeast region. This is an accomplishment of PPSUS in the direction of decreasing regional disparities.

### Health Technology Assessment for SUS

New drugs, equipments, medical devices and procedures are constantly being developed. Ever since 2003, DECIT coordinates the MoH Permanent Working Group on Health Technology Assessment (HTA). In 2005, the MoH appointed a commission to elaborate the National Policy on Health Technology Management (PNGTS), which was approved in 2009 after four years of intense work, reaching a consensus among the representatives of various sectors: health, legal, civil society. The National Commission for Health Technology Incorporation (CITEC) was created in 2006.

In 2004 DECIT started the HTA calls for proposals, totaling 207 projects financed with an investment of around U$10.5 million (U$1.00 = R$1.688 on January 2011). The aim is to impel the SUS towards the regular use of scientific evidence in the decision-making process.

In 2006, DECIT assigned a team specifically dedicated to HTA to subsidize decisions made by the Minister of Health regarding the incorporation of technologies. At this time, the department joined the International Network of Agencies for Health Technology Assessment-INAHTA, a society which brings together 45 agencies from 22 countries. This initiative enabled a greater exchange of information and experiences in the global context. As a result of this effort, Rio de Janeiro, Brazil, was the venue for the 2011 HTAi International Meeting.

Between 2004 and 2010 the Brazilian Cochrane Collaboration was contracted by DECIT to perform 66 systematic reviews; 54 were concluded and most of them were used by decision makers. For instance, the destination of thirty one of these evidence reports (57%) was to support decision making processes: (a) twenty-four were sent to two high level decision units: the National Commission for Health Technologies Incorporation-CITEC and the ministerial group elaborating the Clinical Guidelines; the reviews were incorporated to the body of evidence used by these two units in their decision making process; (b) seven reviews were used straight away to support CITEC decisions. Another seven were employed to compose DECIT's Health Technology Assessment Report.

In 2008, the Brazilian Network for Technology Assessment in Health-REBRATS was established, with the specific goal of supporting the development of priority studies in HTA, to disseminate the studies produced and to establish guidelines for the development and standardization of HTA methods. Presently, there are 44 member institutions in the REBRATS. The network's portal publishes, in Portuguese, over 200 academic studies, systematic reviews and economic evaluations and accepts proposals for new studies (available at: http://www.saude.gov.br/rebrats).

### Research and innovation in neglected diseases-overcoming the 10/90 gap

This is also another strategic research policy. From 2004 to 2009, DECIT and its partners invested around US$70.8 million and launched several calls for proposals, specific for these diseases. The fruitful partnership with the MST and FAPs resulted in an unprecedented pool of financial resources to investigate malaria and dengue fever, via joint calls for proposals in 2009. The first was the Malaria Research Network, with the financial participation of seven states, besides the Federal agencies, investing US$7.5 million. For the Dengue Fever Research Network, co-financing involved 20 states, with US$5.2 million provided by DECIT and partners.

Treatment for most neglected diseases remains an unsolved problem. Recently, a project supported by DECIT showed promising results in the treatment of the cutaneous manifestations of *Leishmania braziliensis*. The randomized controlled trial indicated that after six months of treatment, the oral drug was more effective when compared to the traditional antimony injections regarding clinical remission and treatment compliance, although with a similar incidence of side effects [11].

### Research networks and multicenter studies

In order to optimize resources and results, research networks and multicenter studies are encouraged. The largest cohort of adults in Latin America is the Longitudinal Study of Adult Health, ELSA Brazil, which investigates the determinants, incidence and time course of diabetes and cardiovascular disease in a sample of 15 thousand adults aged between 35 and 74 years old. There are six research centers involved, located in three Brazilian regions: Southeast, South and Northeast. The Ministry of Health and the Ministry of Science and Technology invested US$13.4 million in 2006. More recently, in 2009-2010, DECIT granted around US$4 million to ensure the study's continuity and the preparations for the second wave of data collection. On December 3, 2010, ELSA Brazil reached 102% of the set target, with 15,050 individuals enrolled at baseline to be followed in the subsequent phases.

The National Demography and Health Survey of Women and Children (DHS 2006/7), which aimed to update the health indicators of women and children and its differentials and determinants, was planned and financed by DECIT. The study received an investment of US$4.6 million that was shared with the Coordination of Food and Nutrition at the MoH.

The Study of Cardiovascular Risk in Adolescents (ERICA) began fieldwork in 2010 and aims to estimate the prevalence of diabetes mellitus, obesity, cardiovascular risk factors, inflammatory markers and insulin resistance in 74,000 adolescents 12 to 17 years of age in Brazilian cities with more than 100,000 inhabitants. The study is integrated by 27 research institutions representing all regions of Brazil. In 2008, the MoH and the MST invested US$3.8 million, and in 2010 DECIT provided an additional US$1.2 million to ensure the completion of all laboratory tests.

### Clinical research directed towards SUS needs

Supporting Clinical Research Centers in teaching hospitals in order to answer questions relevant to the SUS was considered essential. Therefore, a joint venture of the MoH and the MST invested US$20.8 million in the National Clinical Research Network (RNPC), launched in 2005. The 19 centers that presented the required criteria, located in all Brazilian regions, were granted funds over a two-year period to refurbish or build research facilities, to acquire equipment and to hire specialized personnel.

Between 2006 and 2008, DECIT and partners invested US$20 million in three calls for proposals to finance RNPC centers to develop studies in areas such as: (1) recombinant human insulin analogue, (2) treatment of leishmaniasis, (3) bariatric surgeries in the SUS, (4) obstructive sleep apnea, (5) osteoporosis, (6) leprosy neuropathy and (7) treatments for prevention of cardiovascular events in hypertensive patients and patients with resistant hypertension.

In 2009, the RNPC was expanded: 13 new centers met the criteria and were eligible to join the network. This allowed for adjusting the role of clinical research to the strategic route of scientific development, accompanying technological advances especially in the health industrial complex.

In 2010, DECIT and CNPq financed projects to conduct phase II or III clinical trials in partnership with private or state-owned companies in order to develop strategic products for the SUS. Three projects were approved: a national ventricular assist device (VAD); skin wound healing action of the proteolytic fraction of a plant extract, and the treatment of venous ulcers with fibrin sealant derived from venoms. The current challenges are related to sustainability in financing and managing the Clinical Research Centers.

### National Network of Cell Therapy-innovation for the future

The seminal project in cell therapy started in 2004: DECIT and partners granted US$7.9 million for "MiHeart", one of the world's largest stem cell clinical trials for cardiopathies [12]. The study organized four branches, dedicated to Chagas heart disease, dilated cardiomyopathy, ischemic heart disease and acute myocardial infarction. More than 30 research centers across the country participate in this study.

In 2005, the Brazilian Congress approved the Bio-safety Act, number 11105/2005, giving breadth to scientific development in the promising area of stem cell therapy and regenerative medicine. There were three calls for proposals, from 2005 to 2008. Eight Cell Technology Centers (CTCs) were supported to produce various types of human stem cells in conditions of good manufacturing practices, being pluripotent stem cells (embryonic and iPSC) or the multipotent (adult hematopoietic, mesenchymal, neural and cardiac). These initiatives led to the constitution of the National Network of Cell Therapy (RNTC) consisting of 52 research groups and eight CTCs. The total investment of DECIT, Finep and the National Bank of Social and Economic Development (BNDES) in this area reached US$49 million. The primary targets are heart disease, chronic degenerative disease, trauma and diseases associated with aging.

Some important results are emerging. A research project funded by DECIT and CNPq in 2005 allowed for the development of a fully national line of embryonic stem cells in São Paulo. Another important result obtained by the same group in Rio de Janeiro, was the development of the first Brazilian strain of induced pluripotent stem cells (iPSC). The Brazilian effort in this novel area of stem cell research was recently classified as innovative by an independent study [13].

### Meeting unexpected challenges: the Influenza A pandemic

In 2009, Brazil was hit by influenza A and the MoH pleaded with the Ministry of Planning for a US$3 million supplementary budget in order to invest in strategic research to face the H1N1 pandemic. DECIT financed several projects in close partnership with the Health Surveillance Secretariat, ranging from the epidemiology of risk factors for disease severity and death, to monitoring genetic mutations of the Influenza A virus circulating in Brazil. Some investments were made in essential innovations for the surveillance of influenza A. Most of the projects have already presented results.

Initially, Brazil, as well as most countries, was totally dependent on inputs and reagents obtained from the US Center for Disease Control for the diagnostic inputs. The "Nationalization of biotechnological products for the molecular diagnosis of Influenza A (H1N1)" project was financed by DECIT in 2009 and six months later the investigators launched the National Diagnostic Kit, promoting access to inputs from molecular biology to achieve diagnostic tests for the Influenza A pandemic virus. Moreover, biotechnological platforms have been deployed in three key national reference laboratories for influenza and in three public health laboratories; equipment maintenance and human resources technical training was also guaranteed ("Pesquisadores brasileiros criam exame molecular que reduz de oito para quatro horas o diagnóstico da gripe A". Correio Brazilense June 21, 2010 <http://www.correiobraziliense.com.br/app/noticia/ciencia-e-saude/2010/06/26/interna_ciencia_saude,199542/index.shtml >)

### Obstacles in the implementation process

To reach the results presented here, many barriers were encountered and partially overcome. The financing mechanisms available at the MoH are not adequate to sponsor research projects and the bureaucracy involved hinders the flow of resources from the federal level to the principal investigators and research institutes. The option to operate the MoH funds through other federal MST agencies, like FINEP and CNPq, on one hand contributed to speed the organization, judgment and disbursement of funds for the Calls for Proposals, but on the other increased the chance that scientists simply forget the origin of the resources (MoH) and acknowledge them as being MST funds.

Inducement mechanisms were not always successful. Sometimes topics of special interest to the MOH were included in Calls for Proposals, but high quality projects were not submitted. Possible explanations to these facts are failures in the research capacity and/or a mismatch between Ministerial priorities and the investigators' research interests. However the events were rare, and did not affect the research agenda fulfillment.

The potential conflicts of interest in the peer review process during the judgment of calls for proposal were overcome by different mechanisms. In the decentralized state bids, research communities are smaller and scientists rather close to each other. In most cases there was a "crossover" mechanism and scientists from other states were summoned to analyze the research proposals. In the national bids CNPq and FINEP have traditional mechanisms to override conflicts of interest. In a few cases scientists working abroad were invited to judge projects.

### Evaluation of the Agenda's fulfillment

The methodology used to assess the NAPHR fulfillment was to compare the investments in research projects with the stated priorities. The analysis was based on data from two DECIT information systems: PesquisaSaude and SisCT (available at: <http://www.saude.gov.br/pesquisasaude > and <http://www.saude.gov.br/sisct >).

The first system was devised to provide transparency and accountability to DECIT processes. It contains the following information about each project: type of call for proposals; title of the project; abstract; keywords; sub-agenda; type and nature of the research; sector of application; the principal investigator (name, contact, affiliation); year; types of support; partnerships and resources approved. The classification of sub-agenda is done by the principal investigator or by DECIT staff.

The SisCT database was built for internal use, to aid the judgment process of grant awards. Once projects are approved and contracts are signed, the SisCT software feeds automatically the "PesquisaSaude" system. The systems are updated daily; the information presented in this commentary was downloaded on January 21, 2011.

Table 2 shows the global investment of DECIT and partners in fostering research and innovation for health between 2004 and 2009. The data is presented by type of call for proposals (state calls, PPSUS, or national public calls) and mode of selection (direct contracts), in which bidding is not required.

**Table 2:** Projects financed and amount invested by the Ministry of Health and partners according to type of call for proposals or mode of selection, Brazil, 2004-2009.

Description	Type of call for proposals or mode of selection			
	**Decentralized calls (PPSUS)**	**National calls for proposals**	**Direct contract of strategic projects**	**TOTAL**

Number of calls for proposals (n)	77	58	77	212
Number of projects financed (n)	1,775	1,705	106	3,586
Resource invested (U$)	75 million	297 million	47 million	419 million
Resource/project invested (U$)	42 thousand	174 thousand	443 thousand	117 thousand

Source: data summaries were calculated by the authors based on information taken from: http://www.saude.gov.br/sisct and http://www.saude.gov.br/pesquisasaude, captured on Jan 21, 2011.

In the period analyzed, there were 135 public calls for proposals, financing 3,480 projects in addition to 106 projects contracted directly with investments of nearly US$420 million. As shown, direct contracting is the least common mode of action and represents about 3% of financed projects and 11% of the resources invested. DECIT disbursed approximately 60% of the total amount. The remaining 40% came from different partners, such as the Ministry of Science and Technology, through the CNPq and FINEP (30% of resources); FAPs with a 6% contribution and other sectors of the Ministry of Health with 4% of the total.

We compared the priorities identified by the 24 NAPHR sub-agendas with the number of projects and the amount invested in each one. The proportions supported were in line with the stated priorities as shown below. Three major criteria guided funding: epidemiologic relevance in the Brazilian context, the needs of the national public health system (SUS) and the development of strategic areas in the national scientific scenario. From 2004 to 2009, DECIT and partners destined US$419 million to support more than 3,500 projects in accordance with the National Agenda, as shown in Table 3. All sub-agenda were funded in different types of calls for proposals with an average investment per project of US$117 thousand. The three sub-agenda receiving the higher amount of resources were "Industrial health complex" (26%), followed by "Clinical research" (18%) and "Communicable diseases" (16%). This reflects the priority given to these areas, considered strategic for innovation and national development; another factor is the need for heavy investments in equipment, laboratories and production plants.

**Table 3:** Projects financed and the amount invested by the Ministry of Health according to the sub-agenda, Brazil, 2004-2009.

Sub-agenda	Number of projects	Amount U$ (×1000)
Afro-Brazilian health	35	1,890
Bioethics and ethics in research	98	1,421
Child and adolescent health	124	5,368
Clinical research	233	74,840
Communicable diseases	672	66,534
Communication and information in health	59	2,981
Demography and health	3	5,605
Elderly health	64	5,347
Epidemiology	67	15,925
Food and nutrition	237	7,315
Health of individuals with disabilities	36	1,597
Health promotion	46	2,174
Health systems and policies	187	7,971
Health technology assessment/economic evaluation	261	18,137
Health, environment, labor and bio-safety	86	6,409
Indigenous health	61	2,030
Industrial health complex	138	108,084
Mental health	153	8,498
Non communicable diseases	379	42,319
Oral health	125	3,331
Pharmaceutical care	163	12,281
Violence, accidents and trauma	98	3,641
Women health	163	9,701
Work and education management in health	98	5,485

Total	3,586	418,883

Source: data summaries were calculated by the authors based on information taken from: http://www.saude.gov.br/sisct, and http://www.saude.gov.br/pesquisasaude, captured on Jan 21, 2011.

The sub-agenda "Bioethics and ethics in research" received the lowest amount of funds and also the lowest funds per project ratio, with an average of US$14 thousand. DECIT initially financed small grants to empower local Ethics Committees of the Research Institutes and Universities, in order to improve quality and promptness in the ethical evaluation of projects according to the National Agenda.

Analyzing the number of projects in each sub-agenda, the figures reinforce that the priorities in health research established by the National Agenda were observed. The sub-agenda with the greatest number of studies approved was, by far, "Communicable diseases". From the total number of projects approved in this sub-agenda, 60.6% (536 studies) were related to neglected diseases. Among the seven neglected diseases elected by the Brazilian government in 2008, dengue fever received the highest amount of resources (19.9%), followed by malaria (17.2%), leishmaniasis (16.1%), tuberculosis (15.2%), leprosy (14.4%), Chagas disease (7.6%), and, lastly, *schistosomiasis *(5.0%).

**Figure 1:**
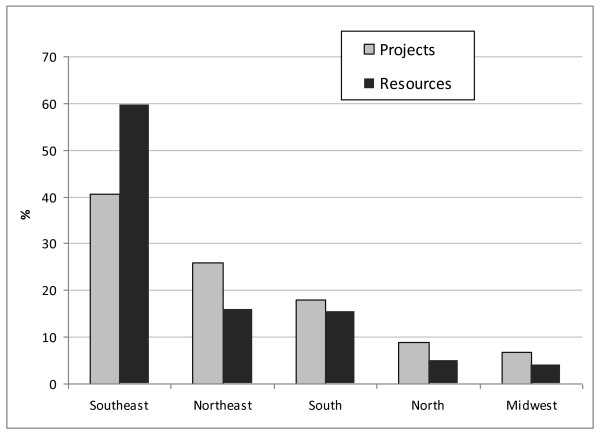
**Proportion of projects approved and amount of resources allocated by the Ministry of Health and partners according to the geographic region**. **Brazil, 2004-2009.** Source: data summaries were calculated by the authors based on information taken from: http://www.saude.gov.br/sisct and http://www.saude.gov.br/pesquisasaude, captured on Jan 21, 2011.

The proportion of resources and projects follows the population distribution in all geographic regions (Figure 1). The Southeast region conducted 40.5% of all projects and detained most of the resources (59.7%). Not surprisingly, the most traditional health research institutes and universities are located in the states of São Paulo and Rio de Janeiro (Southeast region). The Northeast was the second most financed region (both in number of projects and amount of funds) and reflects the result of a governmental policy to integrate and modernize this densely populated area, which used to be the poorest region in the country.

**Figure 2:**
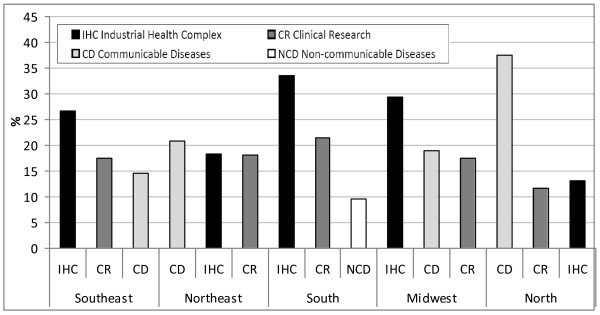
**Proportion of investments by the Ministry of Health and partners in the three most granted sub-agenda, according to geographic region**. **Brazil, 2004-2009.** Source: data summaries were calculated by the authors based on information taken from: http://www.saude.gov.br/sisct and http://www.saude.gov.br/pesquisasaude, captured on Jan 21, 2011.

Figure 2 shows the three sub-agenda that received the greatest amount of funds according to geographic region. There are clear differences that reflect the stage of regional development. In the Southeast, South and Midwest, the most industrialized and developed regions, there was a predominance of funds invested for research in the "Industrial health complex" agenda. On the other hand, in the Northern Amazonian region and in the Northeast, most of the resources were allocated to "Communicable diseases", especially in the North. This data shows that the most advanced technologic and innovative projects lead the agenda mainly in the Southeast, South and Midwest regions. On the other hand, the epidemiologic profile regarding neglected diseases is reflected in the poorest North and Northeast regions, which, alongside the Midwest region, maintain some excellent institutions and university hospitals that have a large tradition in research on neglected diseases.

## Discussion

Although Brazil began the design and implementation of the National Agenda of Priorities in Health Research in 2003 it has done so in accordance with the 'good practice' principles recently published [9]. During the preparatory work we considered the *contextual* factors and the SUS principles; a *comprehensive approach* was developed and employed; the process was *inclusive* and geared towards balanced sex and regional participation [5]; *information gathering* was extensive and resulted in the publication of a book, with contributions from 22 leading scientists [14]; careful *planning for implementation* occurred, as evidenced by the funding allocation policy, transforming priorities into actual research [5]. Deciding on priorities was achieved by the *method* of consensus during the NAPHR Seminar in 2003 and the National Conference of STI in Health in 2004 [5].

Immediately after the priorities were set, the implementation process started [5] facilitated by the political decision to increase by almost five fold the DECIT budget for the period 2004-2007 (for the subsequent budgetary period: PPA 2005-2011, the allocation was further augmented by about 10%). As far as *evaluation*, an internal evaluation is presented here and an external independent evaluation was contracted by DECIT in 2010 and is underway; final results are due in 2012. Health research needs are dynamic and demand new issues to be investigated, implying the need for a periodic revision of the National Agenda. In 2010 the MoH began to update the NAPHR which was widely discussed by investigators, health policymakers and civil society. Preliminary results were presented to the NAPHR Consultative Group Meeting in December 2010, aiming to construct a revised version in 2011/2.

The priority setting process was *transparent *from the start, involving a large number of stakeholders, employing public consultation and afterwards the democratic process of municipal, state and national conferences, the usual way the SUS proceeds. All documents are freely available on the web.

The Peruvian MoH's research portfolio was recently reported [15]; during the period 2004-2008 the budget was U$ 5 million and 182 research projects were approved and funded. Out of the presented research projects, 45% were financed through a competitive fund, 40% were institutional, 9% came from regional/provincial health directions and 5% were collaborative. Compared to the Brazilian MoH experience, presented here, we had a much higher proportion of projects funded by competitive bids (97% of the projects and 89% of the resources invested). The mean amount assigned to each research project in Peru was U$ 33 thousand, whereas in Brazil the average amount invested per project was of U$ 117 thousand. Budget allocation according to study subject was 61% for communicable diseases, 27% for technological development and 12% for non-communicable diseases in Peru. In Brazil the three research subjects receiving more funds were industrial health complex (26%), clinical research (18%) and communicable diseases (16%).

The priority-setting criteria of NIH funding was questioned in the past and greater consideration of disease burden was recommended. Recently the NIH disease funding levels was compared to burden of disease [16]. The authors concluded that in 2006 the levels of disease-specific funding correlated only modestly with US burden of disease (which accounted for 33% of variation) and correlation did not improve compared to 1996 levels. Similar results were obtained for estimates of future US burden, as well as current and future global disease burden.

The Brazilian MoH has some challenges ahead. To work even closer and to prioritize research questions relevant to the National Health Policy, focusing health systems research in the areas of assistance, surveillance, prevention and promotion. To induce investigations aimed to practical applications in the fields of drug development, new technologies and medical devices, as well as in the traditional fields of health research.

## Conclusions

As shown, Brazil implemented and fulfilled the NAPHR financing health research priorities as defined. Furthermore, the funding allocation was in accordance with economic and regional health characteristics and needs. The affirmative policy to combat regional inequities in health research was successful and should remain as a permanent goal. A participatory process was in fact established and this democratic management of health research policy conferred legitimacy, permitting it to become a part of the National Health Policy.

In conclusion, the joint efforts of the Department of Science and Technology with the Ministry of Science and Technology and its institutes (CNPq and FINEP), as well as the State Foundations for Research Support (FAPs) and other departments at the MoH, have achieved and legitimated an unprecedented developmental spurt to support strategic health research. However, nothing of this magnitude would have been achieved without a mature, responsive and committed local scientific community.

We believe our experience is valuable and applicable to other countries. Of course, different settings and local political circumstances will determine the best course of action to follow.

## Competing interests

The authors declare that they have no competing interests.

## Authors' contributions

LMPS, RCBB, SJS, MLM, FTSE, AAT and RG were involved in the development of the NAPHR; RG, LMPS, SJS, MLM, FTSE and APP were responsible for managing research funding in accordance with the NAPHR; SJS, LMPS and GM were in charge of the data management systems. LMPS and ECM analyzed the dataset and prepared the first draft. SJS, RCBB and CAGG contributed to the text and were responsible for the critical review. All authors contributed and approved the final manuscript.
